# The Impact of Male Infertility Research on the International Brazilian Journal of Urology: An Associate Editor’s Overview

**DOI:** 10.1590/S1677-5538.IBJU.2024.9903

**Published:** 2024-03-18

**Authors:** Sandro C. Esteves

**Affiliations:** 1 ANDROFERT - Clínica de Andrologia e Reprodução Humana Campinas SP Brasil ANDROFERT - Clínica de Andrologia e Reprodução Humana, Campinas, SP, Brasil; 2 Universidade Estadual de Campinas Divisão de Urologia Departamento de Cirurgia Campinas SP Brasil Departamento de Cirurgia, Divisão de Urologia - Universidade Estadual de Campinas - UNICAMP, Campinas, SP, Brasil; 3 Aarhus University Faculty of Health Aarhus C Denmark Faculty of Health, Aarhus University, 8000 Aarhus C, Denmark

## INTRODUCTION

The International Brazilian Journal of Urology (IBJU) has played a pivotal role in disseminating knowledge and advancements in the field of urology. Over the period spanning from 2002 to 2023, the journal has made significant strides in addressing various urological topics, including male infertility. In this paper, we aim to shed light on the substantial contribution of male infertility research to the IBJU over the years.

## MALE INFERTILITY IN IBJU

**Figure 1:**
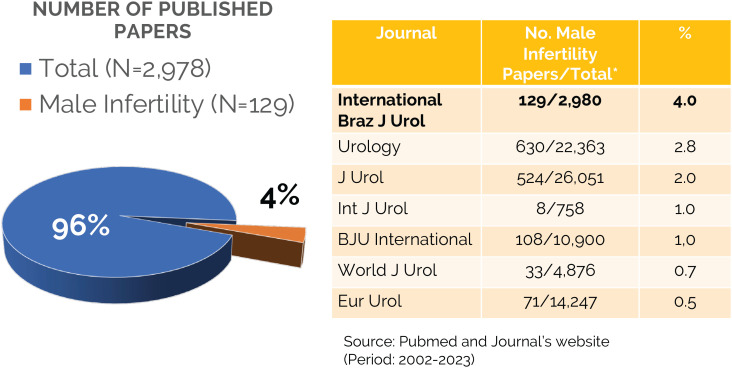
Male infertility research bibliometrics in the International Brazilian Journal of Urology and its comparison with other major urological journals.

During the aforementioned period, the IBJU published a total of 129 articles dedicated to the key area of male infertility. When viewed in the context of the journal’s comprehensive coverage, this accounts for 4% of the total articles published (Figure-1). Although this percentage may appear relatively modest compared to other subspecialty urological areas, it stands as a noteworthy achievement for the IBJU when compared to the relative contribution of ‘male infertility’ in high-impact factor journals in the urological field.

## COMPARISON WITH OTHER JOURNALS

To provide perspective, let us compare this figure to some other prominent journals in the field of urology. Urology, J Urol, Int J Urol, BJU International, World J Urol, and Eur Urol reported male infertility contributions ranging from 0.5% to 2.8% during the same period. The IBJU’s commitment to male infertility research is evident, surpassing many of its counterparts in this regard.

## CITATIONS AND IMPACT

The impact of male infertility research in the IBJU is further demonstrated by the citation numbers. The 129 articles received a total of 3474 citations (range: 0-314), with an average of 27 citations per article. This signifies the importance and influence of the research conducted within this subarea. Notably, the top-cited articles often involved the direct or indirect participation of the journal’s editors, including the Chief Editor, Associate Editor, and Section Editor, underscoring their commitment to advancing male infertility knowledge (Table-1).

**Table 1:** Most cited papers in the area of male infertility in the International Brazilian Journal of Urology during the period of 2002-2023.

Rank	Paper title	Article type	Year	Citation Number	Authors	Participation of Editors or Editorial Board*
1	Unexplained male infertility: diagnosis and management		2012	314	Hamada et al.	X
2	Clinical relevance of oxidative stress and sperm chromatin damage in male infertility: an evidence-based analysis		2007	313	Cocuzza et al.	
3	Cell phones and male infertility: a review of recent innovations in technology and consequences		2011	211	Agarwal et al.	X
4	Clinical relevance of routine semen analysis and controversies surrounding the 2010 World Health Organization criteria for semen examination		2014	180	Esteves	X
5	Sperm retrieval techniques for assisted reproduction		2011	178	Esteves et al.	X
6	Novel concepts in male infertility		2011	148	Esteves & Agarwal	X
7	Definition and current evaluation of subfertile men		2006	143	Shefi & Turek	
8	Laparoscopic diagnosis and treatment of nonpalpable testis		2008	119	Tavilani et al.	
9	Recovery of spermatogenesis after microsurgical subinguinal varicocele repair in azoospermic men based on testicular histology		2005	112	Esteves & Glina	X
10	The effect of adjuvant vitamin C after varicocele surgery on sperm quality and quantity in infertile men: a double-blind placebo controlled clinical trial		2015	113	Cyrus et al.	
11	Impact of infection on the secretory capacity of the male accessory glands		2009	110	Marconi et al.	
12	Sperm defect severity rather than sperm Source is associated with lower fertilization rates after intracytoplasmic sperm injection		2008	100	Verza Jr. & Esteves	X
13	Successful treatment of unilateral cryptorchid boys risking infertility with LH-RH analogue		2008	84	Hadziselimovic	
14	Laparoscopic diagnosis and treatment of nonpalpable testis		2008	84	Denes et al.	
15	Wet heat exposure: a potentially reversible cause of low semen quality in infertile men		2007	72	Shefi et al.	
16	Evaluation of acrosomal status and sperm viability in fresh and cryopreserved specimens by the use of fluorescent peanut agglutinin lectin		2007	75	Esteves et al.	X
17	Influence of antisperm antibodies in the semen on intracytoplasmic sperm injection outcome		2007	63	Esteves et al.	X
18	Chromosomal and molecular abnormalities in a group of Brazilian infertile men with severe oligozoospermia or non-obstructive azoospermia attending an infertility service		2011	63	Mafra et al.	
19	Applied anatomic study of testicular veins in adult cadavers and in human fetuses		2007	62	Favorito et al.	X
20	Apoptotic markers in semen of infertile men: Association with cigarette smoking		2011	57	El-Melegy et al.	
21	Male fertility potential alteration in rheumatic diseases: a systematic review		2016	57	Tiseo et al.	
22	Male infertility in spinal cord trauma		2005	54	Utida et al.	

* Direct or indirect involvement in attracting the submission to the journal.

## INNOVATION AND THE ‘PATIENT CORNER’

Male infertility research has not only contributed to the citation count but has also sparked innovation within the IBJU. The creation of the ‘Patient Corner’ is a prime example (1). This section features short articles written in layman’s terms, addressing specific urological conditions to serve the patient community. The inaugural article in this section was dedicated to varicocele, a common treatable condition affecting male infertility (2). The IBJU welcomes articles of this nature, along with reviews, original papers, surgical techniques, radiology reports, videos, and letters to the editor.

## LOOKING FORWARD

As we reflect on the past two decades, it is evident that male infertility research has become an integral part of the International Brazilian Journal of Urology. We anticipate a bright future for this subarea within the journal and invite the global urological community to embrace the remarkable growth that the IBJU has witnessed in recent years. Together, we can continue to advance the field of male infertility and provide valuable insights for both clinicians and patients alike.

## CONCLUSION

The contribution of male infertility research to the IBJU is substantial and reflects the journal’s commitment to advancing knowledge in urology. Despite its modest percentage compared to the contribution of other urological specialties, the impact and innovation in this subarea are undeniable. With the support of the urological community, the IBJU is poised to further elevate the field of male infertility research in the coming years.
